# Characterization of a novel Fluoride resistant bacterial isolate and its capability of Fluoride bioremediation

**DOI:** 10.3934/microbiol.2022031

**Published:** 2022-11-23

**Authors:** M Thirumala, E Sai Krishna, P Sindhu Priya, S Vishnuvardhan Reddy

**Affiliations:** 1 Microbial Ecology Laboratory, Department of Biochemistry, UCS, Mahatma Gandhi University, Anneparthy, Yellareddygudem (PO), Nalgonda-508254, Telangana, India; 2 Microbztech Labs Pvt. Ltd., Cherlapally, Nalgonda-508001, Telangana, India

**Keywords:** defluoridation, fluoride resistance, *Bacillus* sp., sodium fluoride, 16S rDNA

## Abstract

A Gram positive rod shaped bacterium designated as isolate H1 with Fluoride resistance up to 4 g/L sodium fluoride (NaF) in LB (Luria-Bertani) agar was isolated from a ground water sample of Narketpally area, Nalgonda district, Telangana, India. The colonies of isolate H1 were off white in color. Growth patterns of isolate H1 were observed at two different concentrations, 100 and 250 ppm, of NaF and also without NaF in the medium. In cases where NaF was present in the media, the lag phases of the growth curves were extended when compared to the absence of NaF. Optimum pH required for the organism's growth was 8. Isolate H1 required a temperature of 37 °C with 150 rpm and 2% NaCl for its optimal growth in the medium without NaF. Meanwhile, isolate H1 could thrive in a diverse pH range, i.e., pH 5–10, and at an NaCl concentration of up to 11% in the medium with NaF. Based on morphological, biochemical and molecular characterization, isolate H1 was identified as belonging to the genus *Bacillus*. It showed 98.47% 16S rDNA gene sequence similarity with *Bacillus australimaris* NH71_1^T^. Isolate H1 showed high fluoride removals of 22.5% and 38.2% with 100 and 250 mg/L of NaF in the LB broth when incubated at pH 8 and a temperature of 37 °C with 150 rpm for 3 day. Hence, this organism could be a promising isolate to apply for defluoridation of ground water in fluoride contaminthe ated areas.

## 1. Introduction

Fluorine amounts to around 0.3 g/kg of the total earth's crust, existing in the forms of fluoride in fluorspar, fluorapatite and cryolite minerals [1]. According to the World Health Organization (WHO), the permissible limit for fluoride in drinking water is 1.5 mg/L [2]. Fluoride has an important role as an anti-caries agent. It not only protects dental hard tissue but also inhibits bacterial growth and metabolism with acidogenicity, acidurance and adherence to the tooth surface. To prevent the destructive effects of fluoride, oral bacteria are capable of developing resistance to fluoride with changes in their genomes [3]. Different fluoride-resistant organisms are able to grow in an environment with fluoride concentration in the range of 400–1,000 ppm (21.1–52.6 mM).

Bacteria could gain different abilities to withstand environmental changes like certain levels of fluoride [4], and they have a major role in bioaccumulation, biosorption and biotransformation [5]. When the fluoride ion is present inside the bacterial cells, it can directly or indirectly inhibit some of the metabolic enzymes, namely, Enolase, Urease and F-ATPase, which can cause decreases in bacterial growth and metabolism [6]–[10]. Three of the fluoride resistant bacteria (200 mM) isolated from ground waters in selected villages at Dindigul district, Tamilnadu, India, were resistant to antibiotics such as Amoxicillin, Ampicillin, Chloramphenicol, Kanamycin and Streptomycin [11].

Removal of toxic compounds from the environment can be accomplished by some microorganisms [12],[13], nanoparticles [14] and some agricultural wastes [15]. Different fluoride resistant organisms could remove different percentages of fluoride from media [16]–[21].

To understand the mechanism of fluoride resistance in fluoride-resistant *Streptococcus mutans* strain (C180-2FR), whole-genome shotgun (WGS) sequencing was done [22]. Effects of pH on expression of NaF resistance in *Streptococcus mutans* was observed, in which at lower pH values mutants showed resistance to fluoride than the parent strain [23]. In fluoride resistant strain c180-2FR, two glycolytic enzymes were undergone mutations they were pyruvate kinase and enolase. Mutations were also observed in promoter *mutp*, with which upregulated expression of downstream fluoride antiporters was observed [24]. The decreased enolase activity was not always associated with decreased *S. mutans* growth in the presence of NaF [25].

Fluoride contamination in ground water is a major issue in some areas of Nalgonda district. Intake of water with high fluoride content leads to health issues like dental and skeletal fluorosis. To avoid such health problems, defluoridation should be done to the ground water before consumption. Wild strains with the capacity of bioremediation of fluoride exist in nature, which can be helpful to mankind in this regard. In this report, a high fluoride resistant isolate H1 was isolated from ground water sample of Narketpally area, Nalgonda district, Telangana, India, and was characterized and checked for its bioremediation activity.

## 2. Materials and methods

### 2.1. Sample collection

Ground water samples were collected from various hand and bore pumps at various seasonal intervals from in and around Narketpally area (17°19′N 79° 20′E), Nalgonda district, Telangana, India.

### 2.2. Isolation and adjustment of bacterial isolates on media with NaF

High fluoride containing ground water samples of Narketpally area, Nalgonda district, Telangana, India, were used for serial dilution and then plated onto Luria–Bertani (LB) agar medium consisting of (g/L-1) Casein Enzyme Hydrolysate (Tryptone) (10), Yeast Extract (5), NaCl (5) and Agar (15), with final pH 7.0 ± 0.2. Plates were then incubated at 37 °C for 24 h. Bacterial isolates were randomly chosen and purified. After purification of bacterial isolates on LB agar, they were further plated onto the media with various NaF concentrations (i.e., 500, 1000, 1500, 2000, 2500, 3000, 3500, 4000 and 4500 mg/L) in a stepwise manner following initial growth in broth at 150 rpm, and then the organisms were plated onto the agar. Plates were incubated at 37 °C for 24 h. Exposing isolates to high concentrations of fluoride suddenly may cause the inhibition of growth, so the organisms were grown initially with low concentrations then transferred to high concentrations of NaF, which may help the isolates to adapt to those environments [26].

### 2.3. Characterization of isolate H1

High fluoride resistant organism H1 was characterized morphologically, physiologically and biochemically.

Under morphological characteristics: Colony morphology and Gram's test were performed. Effects of temperature and pH on the optimal growth of Fluoride resistant organism H1 were tested in LB broth. Growth of the fluoride resistant organism H1 was checked in LB broth at different temperatures (such as 4 °C, 25 °C, 37 °C and 45 °C) in an incubator for 48 h. Growth of the fluoride resistant organism H1 was also checked in LB broth at different pH values, ranging from 5 to 10 in an incubator for 48 h at 37 °C with NaF and without NaF in the media separately. Effect of NaCl on the growth of the H1 organism was examined using different concentrations of NaCl (2% to 12%) in the LB broth.

Spectrophotometer 104 (Systronics) was employed to measure the growth of the fluoride resistant organism H1 both in presence (4 g/L NaF) and absence of NaF. Growth of isolate H1 was measured in terms of Optical density (OD) at 600 nm from 24 h to 126 h. Then, the OD values were plotted against time to determine the growth curve of the isolate H1. Along with the growth pattern of isolate H1, a change in the pH of the medium was also recorded for around 5 days. Biochemical tests like the Methyl Red test, Voges-Proskauer test and Oxidase, Catalase and Nitrate Reduction tests were performed. Himedia Hicarbo kit was employed to study carbohydrate utilization of high fluoride resistant isolate H1.

#### 2.3.1. Methyl Red test

This test was conducted to analyze the capability of the isolate for its performance of mixed acid fermentation as modified by MacFaddin [27]. In brief, 100 µL of H1 inoculum was inoculated in a Methyl Red Voges-Proskauer (MRVP) broth containing tube and incubated at 37 °C for 24 h. After incubation, five drops of methyl red were added to the tube, and the result was recorded. A color change to red indicates the presence of mixed acid fermentation.

#### 2.3.2. Voges-Proskauer test

This test was performed to analyze the capacity of the organism, whether it produces the 2, 3 butanediol as a fermentation product from Glucose. As 2, 3 butanediol cannot be easily detected, acetoin, the intermediate in this pathway, can be targeted in this test. Isolate H1 inoculum was inoculated in the MRVP broth containing tube and incubated at 37 °C for 24 h. After incubation, five drops of Barritt's A reagent followed by five drops of Barritt's B reagent were added. Then the tube was kept in slant position for half an hour. A color change to red indicates a positive test [27]

#### 2.3.3. Catalase test

This test was carried out using the Evans and Kloos method [28]. In this method, a sterile loop was used to collect a 24 h-old colony of H1 isolate from LB agar medium. It was placed on a clean slide, and smear was prepared. Then, a single drop of 3% H_2_O_2_ was added to the smear. Formation of bubbles indicates the presence of catalase.

#### 2.3.4. Oxidase test

Fresh colony of H1 isolate using sterile loop was rubbed onto a moistened strip, which has Oxidase reagent (1% N,N,N,N,-tetramethyl p-phenylenediamine dihydrochloride) impregnated on it. This chemical replaces O_2_ as a recipient for the electrons from the enzyme cytochrome oxidase. The additional electrons change the oxidase reagent from colorless to purple. No change in color indicates a negative test.

#### 2.3.5. Nitrate reduction test

This test was performed to determine the capacity of the organism, whether it reduces nitrate to nitrite, utilizing the nitrate reductase enzyme. For this test, Nitrate broth was prepared with potassium nitrate and other nutrients, and then H1 organism was inoculated into it. Uninoculated nitrate broth was used as a control. Both uninoculated and inoculated tubes were incubated at 37 °C for 24 h. After incubation, one drop of each of reagent A (0.8 g of sulfanilic acid in 100 mL of 30% acetic acid) and reagent B (500 mg of N, N-dimethyl-1-naphthylamine in 100 mL of 30% acetic acid) was added. After addition of a pinch of powdered zinc, results were recorded. A change in color to red after the addition of both A and B reagents, followed by its disappearance on addition of zinc, indicates the positive test result. Appearance of no color after adding both reagents followed by a change in color to red, on addition of zinc, indicates a negative result.

Carbon substrate utilization tests employing Hicarbo kit and antibiotic sensitivity tests (with twelve different antibiotic strips on LB medium) were performed on fluoride resistant isolate H1.

### 2.4. Molecular Identification of Fluoride resistant isolate H1

Fluoride resistant isolate H1 was identified using 16s rDNA gene amplification, sequencing [29] and construction of phylogenetic tree. DNA was extracted by using the heat treatment technique. Then, Polymerase chain reaction (PCR) was performed in thermocycler to amplify the 16s DNA gene by using forward and reverse primers [30] 27F (5′-AGAGTTCCTGCTGCAG-3′) and 1492R (5′-GGTTACCTTGACTTTT-3′), respectively [31]. The PCR program applied here was as follows: initial denaturation at 95 °C for 5 min, then denaturation at 95 °C for 1 min, followed by annealing of primers at 55 °C for 1 min and extension at 72 °C for 2 min. PCR was performed for 30 cycles, and final extension was carried out at 72 °C for 15 min. Amplicons were maintained at 4 °C. A 1.5 kb size PCR product was initially analyzed using Agarose gel electrophoresis, and then it was purified and sequenced. To identify isolate H1, EZBiocloud public data and analytics portal were used (https://www.ezbiocloud.net/apps). The 16s rDNA gene sequence of high Fluoride resistant isolate H1 was submitted to the NCBI Gene bank, and its accession number was sought. Mega 11 software was used for its phylogenetic analysis.

### 2.5. Bioremediation of fluoride by high fluoride resistant isolate H1

To determine the fluoride removal activity of the high fluoride resistant isolate H1, it was inoculated and incubated at a temperature of 37 °C with 150 rpm and pH 8 in 250 mL LB broth with 100 and 250 mg/L fluoride concentrations (NaF), separately. Remaining Fluoride content in the media was determined every day till 8 days. For this, a 20 mL culture sample was taken out aseptically every day, from which 5 mL was used to take optical density readings at 600 nm to analyze the growth of the H1 organism, and 15 mL of Sample was used to find out the bioremediation activity of the isolate H1 for a period of 8 days. To analyze the bioremediation activity, the 15 mL sample was centrifuged at 4500 rpm for 15 min. Then, supernatant was used for analyzing remaining fluoride concentration with a fluoride ion selective electrode [17], where TISAB III (Total ionic strength adjustment buffer III solution) reagent was mixed with supernatant sample in 1:1 ratio. These tests were performed in triplicate. Taking the OD values and fluoride removal test results on same days helps to establish a link between growth phase and its capability of Fluoride removal.

## 3. Results

### 3.1. Isolation and adjustment of bacterial isolates on media with NaF

The ground water samples of Narketpally area, Nalgonda district, Telangana, India, were inoculated on LB agar with different concentrations of fluoride to purify high fluoride resistant organisms. Two such isolates, H1 and H2, were purified and could resist the fluoride concentrations of 4.8 and 4.4 g/L, respectively, in broth. Meanwhile, isolates H1 and H2 could resist the fluoride concentration on agar plates was 4 and 2.8 g/L, respectively. In this report, a study on H1 isolate is only mentioned.

### 3.2. Characterization of isolate H1

**Figure 1.**
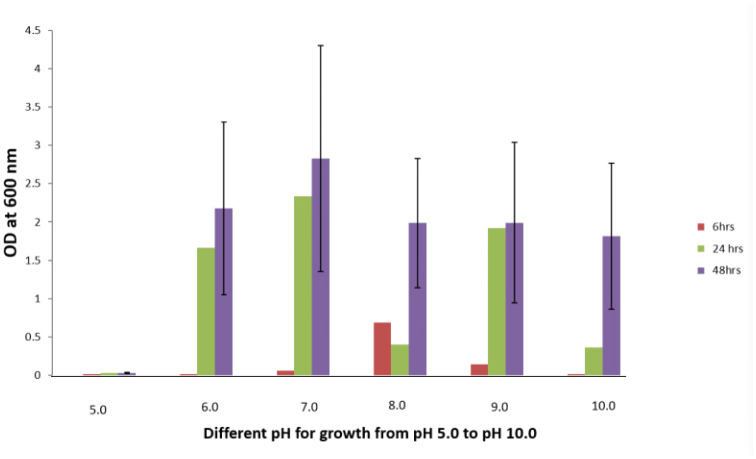
Effect of pH on the growth of fluoride resistant organism H1 in the LB medium without NaF.

**Figure 2.**
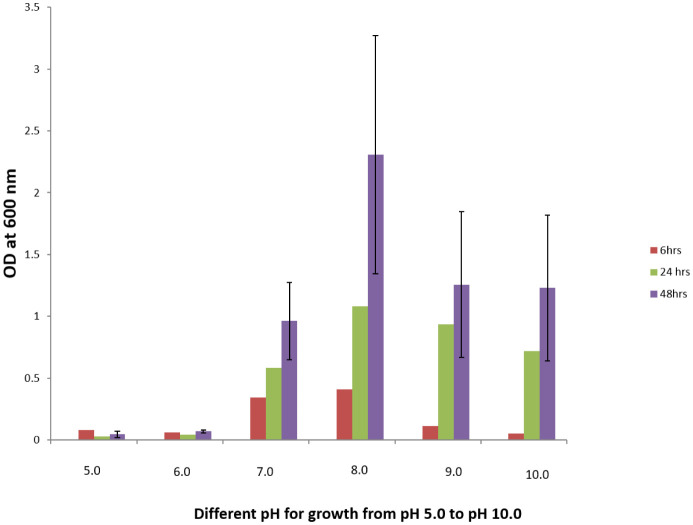
Effect of pH on the growth of fluoride resistant organism H1 in the LB medium with NaF (4 g/L).

Isolate H1 colonies were off white in color, cells were rod shaped, and they showed a Gram positive test. When grown at different temperatures (like 4 °C, 25 °C, 37 °C and 45 °C) in an incubator for 48 h, the fluoride resistant organism H1 showed 37 °C as its optimum temperature required for its proper growth. Isolate H1 was tested for optimum pH required for its growth in the presence and absence of NaF (4 g/L) in the medium for 48 h. The optimum pH showed for the growth of the organism H1 was 7 without the presence of NaF in the medium (Figure 1). However, the optimum pH showed for the growth of isolate H1 in the presence of NaF (4 g/L, i.e., the organism's NaF resistance limit in the agar) was 8 (Figure 2). When H1 organism was grown with different concentrations of NaCl, 2% showed as the optimum NaCl concentration for its proper growth.

**Figure 3.**
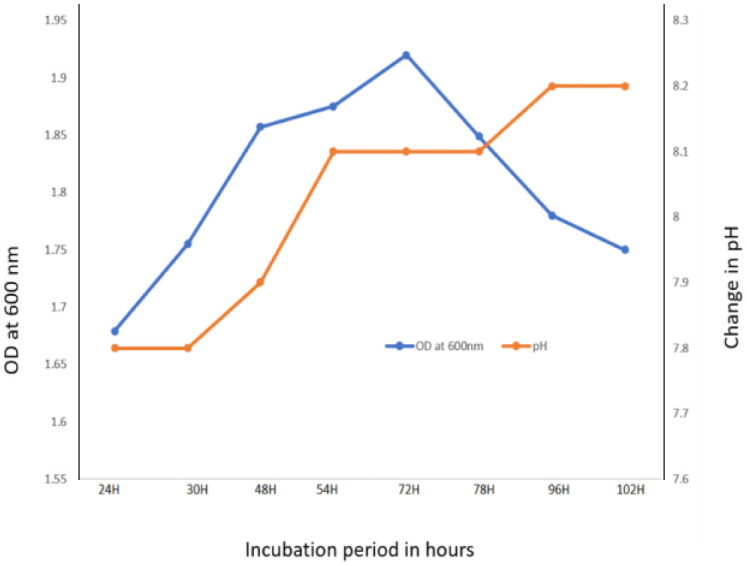
Effect on medium's (without NaF) pH by Fluoride resistant isolate H1.

**Figure 4.**
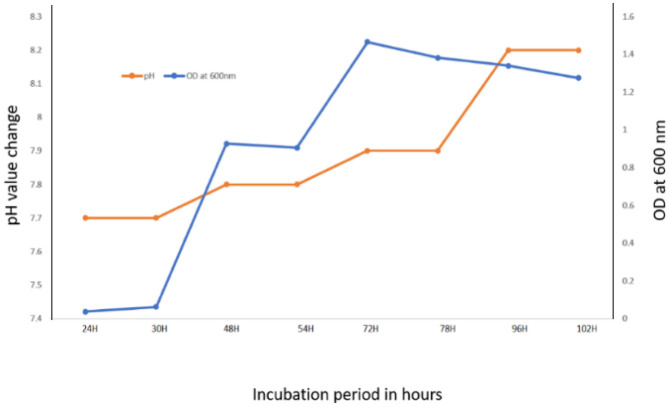
Effect on medium's (with NaF (4g/L)) pH by Fluoride resistant isolate H1.

The growth curve of the organism H1 was determined by taking OD values in a spectrophotometer at 600 nm at regular intervals from 24 h to 102 h. Isolate H1 was showing exponential phase at 72 h both in the absence of NaF and presence (4 g/L NaF) in the medium. The growth of isolate H1 was faster in the media without NaF when compared to with NaF. In both the absence and presence of NaF (4 g/L) in LB broth with isolate H1, a rise in the medium's pH was seen as the incubation time increased. Initial pH of 7.5 adjusted in the medium was gradually increased to 8.2 after 96 h of incubation at 37 °C. Medium's pH was increasing slowly when NaF was present in the medium than without NaF. After that, no further considerable change in pH was observed (Figures 3 and 4).

### 3.3. Identification of fluoride resistant isolate H1

Isolate H1 was showing negative results for Methyl Red and Voges-Proskauer tests, butOxidase, Catalase and Nitrate reduction tests were positive. Different carbohydrate utilization tests were performed. Isolate H1 could efficiently utilize Fructose, Dextrose, Galactose, Mannitol, Adonitol and Arabitol as carbon substrates. Whereas, Lactose, Sucrose, L-Arabinose, Glycerol, Dulcitol, Sorbitol, Xylitol, D-Arabinose, Malonate and Sorbose did not support the growth of isolate H1. Hydrolytic activity of ONPG was absent, and Esculin hydrolysis was positive by this isolate. Citrate could be utilized by the isolate H1 but not α-Methyl-D-Glucoside. Other substrates which feebly support the growth of the isolate H1 are also mentioned in Table 1.

**Table 1.** Different Carbohydrates' utilizations by Fluoride resistant isolate, H1.

S. No	Carbohydrate utilization	Result
1	Lactose	-
2	Xylose	+
3	Maltose	+
4	Fructose	+++
5	Dextrose	+++
6	Galactose	+++
7	Raffinose	+
8	Trehalose	+++
9	Melibiose	+
10	Sucrose	-
11	L-Arabinose	-
12	Mannose	++
13	Inulin	++
14	Sodium gluconate	++
15	Glycerol	-
16	Salicin	++
17	Dulcitol	-
18	Inositol	++
19	Sorbitol	-
20	Mannitol	+++
21	Adonitol	+++
22	Arabitol	+++
23	Erythritol	++
24	α-Methyl-D-glucoside	-
25	Rhamnose	++
26	Cellobiose	++
27	Melizitose	-
28	α-Methyl-D-Mannoside	-
29	Xylitol	-
30	ONPG	-
31	Esculin	+
32	D-Arabinose	-
33	Citrate	+
34	Malonate	-
35	Sorbose	-

Note: + = positive result; ++/+++ = signs given based on the increased intensity of color formed for positive test; - = Negative result

Out of twelve antibiotics tested for sensitivity, isolate H1 showed resistance to five antibiotics: Chloramphenicol (30 mcg), Erythromycin (15 mcg), Penicillin (10 mcg), Nalidixic acid (10 mcg) and Ampicillin (10 mcg). Meanwhile, isolate H1 was inhibited by Tetracycline (30 mcg), Amikacin (30 mcg), Kanamycin (30 mcg), Vancomycin (30 mcg), Gentamycin (10 mcg), Streptomycin (10 mcg) and Ciprofloxacin (5 mcg) (Table 2).

**Table 2.** Antibiotic sensitivity tests of Fluoride resistant isolate, H1.

S.No.	Antibiotic	Result
1	Amp10	R
2	TE30	S
3	AK30	S
4	NA30	R
5	P10	R
6	E15	R
7	K30	S
8	VA30	S
9	GEN10	S
10	S10	S
11	CIP5	S
12	C30	R

Note: R = Resistant; S = Sensitive; Amp10 = Ampicillin; TE30 = Tetracycline; AK30 = Amikacin; NA30 = Nalidixic acid; P10 = Pencillin-G; E15 = Erythromycin; K30 = Kanamycin; VA30 =Vankomycin; GEN10 = Gentamicin; S10 = Streptomycin; CIP5 = Ciprofloxacin; C30 = Chloramphenicol.

**Figure 5.**
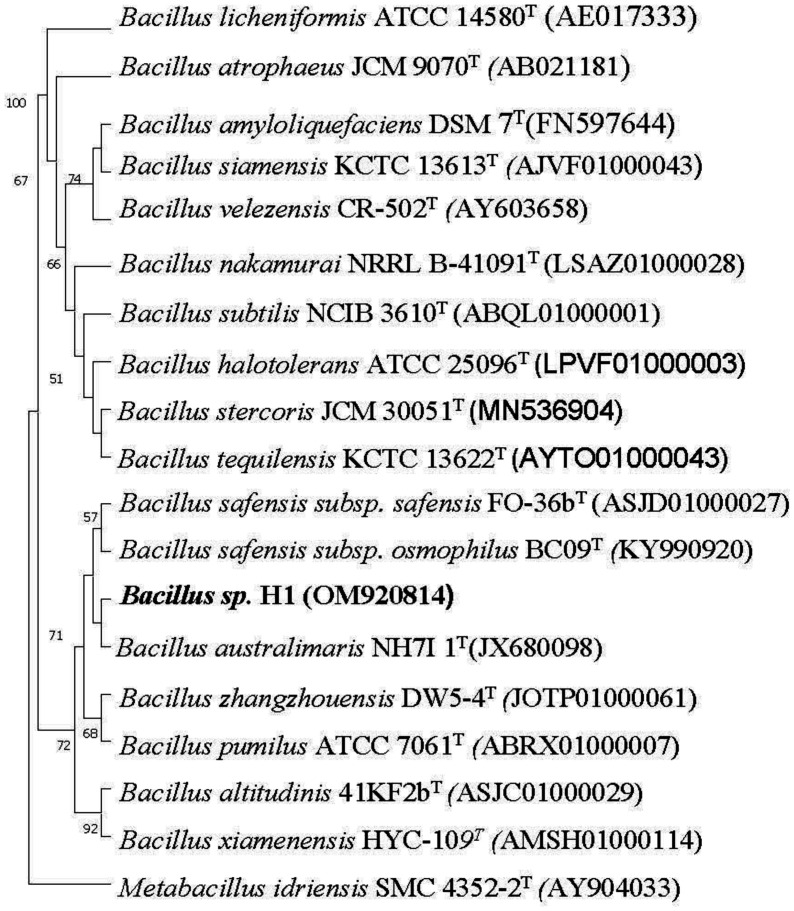
Neighbor-Joining Tree of the isolate H1 along with its closely related type strains.

The 16s rDNA gene sequence of the isolate H1 was uploaded onto the 16S and genome sequence database EzBiocloud. This fluoride resistant isolate H1 was identified as belonging to the genus *Bacillus*. It was showing 98.47% 16S rDNA gene sequence similarity with *Bacillus australimaris* NH71_1^T^. The 16S rDNA gene sequence of high fluoride resistant isolate H1 was submitted to the NCBI Gene bank, and the accession number sought was OM920814. The Neighbor-Joining tree was constructed as part of Phylogenetic analysis employing Mega 11 software (Figure 5).

### 3.4. Bioremediation of Fluoride by high Fluoride resistant isolate H1

**Figure 6.**
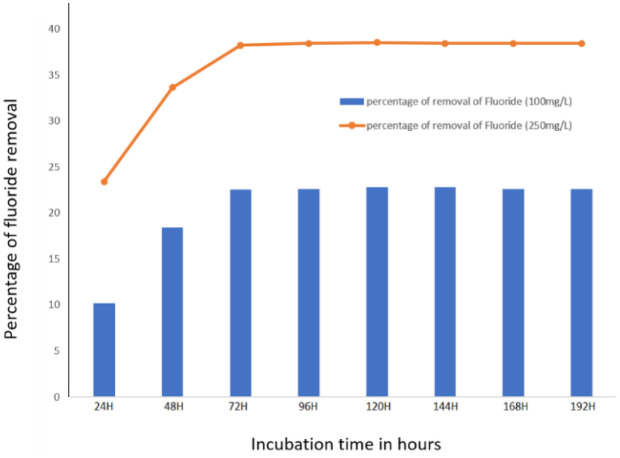
Defluoridation by Fluoride resistant isolate H1.

Defluoridation by Fluoride resistant organism H1 was efficient when Fluoride concentration was 250 mg/L in the medium than 100 mg/L. The Fluoride resistant isolate H1 could remove 38.2% of Fluoride from the LB broth (with initial 250 mg/L NaF) after 72 hours of incubation. However, when the removal of the Fluoride concentration was lower, i.e., 22.5% only with 100 mg/L NaF (Figure 6), there was no considerable change in Fluoride concentration, which must be due to bacterial cell count reduction.

## 4. Discussion

Fluorine is an important 13^th^ most prevalent element of the earth's crust [1]. Microorganisms that are omnipresent have great potential to resist various environmental changes. Similarly, the microbes have the ability to withstand certain levels of Fluoride [4], which they accomplish by different methods, viz., bioaccumulation, biosorption and biotransformation [5]. Usually, the ionic form of Fluoride inside the microbial cells inhibits directly or indirectly various metabolic enzymes, thus negatively affecting their growth and metabolism [6]–[10].

Microorganisms which have the capacity to remove Fluoride from the water exist in nature. Five Fluoride resistant organisms were isolated, and *Pseudomonas aeruginosa* could achieve the removal of 22.1% of Fluoride [16]. Strain RH5 achieved 25.7% Fluoride removal from the media with pH 7 and temperature 30 °C after 8 day of incubation [17]. Two Fluoride tolerant bacterial strains, *Bacillus sp*. KT201599 and KT201600, were isolated from the mid-gut of third instar larvae of *Drosophila melanogaster*, which could survive at 2000–2500 µg/mL sodium Fluoride (NaF) concentrations and have the capability to remove Fluoride from the media by 16.66 and 24.71%, respectively [18]. Meanwhile, Fluoride resistant isolate H1 with high Fluoride resistance of 4 g/L in LB broth, purified in this study, showed 38.2% Fluoride removal activity from the media with NaF concentration of 250 mg·L^−1^ after incubation of 72 h at temperature 37 °C and initial pH 7.

In another study, Fluoride absorption efficiency of two highly Fluoride ion tolerant bacterial strains, *Bacillus cereus* FT1 and *Bacillus marisflavi* FT2, isolated from soil samples collected from Fluoride endemic areas of Birbhum district (Rampurhat block II) of West Bengal, India, were not affected at lower Fluoride concentrations (10–100 ppm). However, at higher Fluoride concentration (730 ppm), the absorption efficiency was significantly increased compared to the control strain (*B. licheniformis* ONF2). The concentrations of Fluoride in the culture media of *B. cereus* FT1 and *B. marisflavi* FT2 were reduced from 730 ppm to 570 ppm and 730 ppm to 580 ppm, respectively, after 72 h of incubation [19].

Interestingly, a Fluoride-tolerant halophilic *Bacillus flexus* NM25 isolated from Fluoride-affected soil in Birbhum District, West Bengal, India, was reducing Fluoride concentration up to 67.45%, and it could tolerate 1,500 ppm of Fluoride in brain-heart infusion agar medium [20]. Meanwhile, *Acinetobacter* sp. isolate could eliminate 57% of Fluoride at 35 °C after 10 h of incubation with 40 mg/100 mL biosorbent and 7.5 pH [21].

Hence, it is of great importance to isolate, purify and characterize wild microorganisms with Fluoride resistance capability and bioremediation activity, so new insights into the metabolism of Fluoride resistance bacteria could be possible, along with their exploitation for human welfare.

## 5. Conclusion

In the present study, isolate H1 with high Fluoride resistance of 4 g/L in LB broth was purified from high Fluoride containing ground water samples of Narketpally area, Nalgonda district, Telangana, India. It showed 38.2% defluoridation activity after 72 h of incubation in the medium with 250 mg/L NaF at 37 °C with initial pH 7. Though the isolate H1 showed promising results of Fluoride bioremediation, a further study needs to be conducted in this regard to exploit the capability of this organism for biological removal of Fluoride from the contaminated ground water samples.
